# Thermal Reaction Norms and the Scale of Temperature Variation: Latitudinal Vulnerability of Intertidal Nacellid Limpets to Climate Change

**DOI:** 10.1371/journal.pone.0052818

**Published:** 2012-12-21

**Authors:** Simon A. Morley, Stephanie M. Martin, Robert W. Day, Jess Ericson, Chien-Houng Lai, Miles Lamare, Koh-Siang Tan, Michael A. S. Thorne, Lloyd S. Peck

**Affiliations:** 1 British Antarctic Survey, Natural Environment Research Council, High Cross, Cambridge, United Kingdom; 2 Eynesbury, St. Neots, United Kingdom; 3 Zoology Department, University of Melbourne, Parkville, Australia; 4 Department of Marine Science, University of Otago, Dunedin, New Zealand; 5 Tropical Marine Science Institute, National University of Singapore, Singapore; The Australian National University, Australia

## Abstract

The thermal reaction norms of 4 closely related intertidal Nacellid limpets, Antarctic (*Nacella concinna*), New Zealand (*Cellana ornata*), Australia (*C. tramoserica*) and Singapore (*C. radiata*), were compared across environments with different temperature magnitude, variability and predictability, to test their relative vulnerability to different scales of climate warming. Lethal limits were measured alongside a newly developed metric of “duration tenacity”, which was tested at different temperatures to calculate the thermal reaction norm of limpet adductor muscle fatigue. Except in *C. tramoserica* which had a wide optimum range with two break points, duration tenacity did not follow a typical aerobic capacity curve but was best described by a single break point at an optimum temperature. Thermal reaction norms were shifted to warmer temperatures in warmer environments; the optimum temperature for tenacity (T_opt_) increased from 1.0°C (*N. concinna*) to 14.3°C (*C. ornata*) to 18.0°C (an average for the optimum range of *C. tramoserica*) to 27.6°C (*C. radiata*). The temperature limits for duration tenacity of the 4 species were most consistently correlated with both maximum sea surface temperature and summer maximum in situ habitat logger temperature. Tropical *C. radiata*, which lives in the least variable and most predictable environment, generally had the lowest warming tolerance and thermal safety margin (WT and TSM; respectively the thermal buffer of CT_max_ and T_opt_ over habitat temperature). However, the two temperate species, *C. ornata* and *C. tramoserica*, which live in a variable and seasonally unpredictable microhabitat, had the lowest TSM relative to in situ logger temperature. *N. concinna* which lives in the most variable, but seasonally predictable microhabitat, generally had the highest TSMs. Intertidal animals live at the highly variable interface between terrestrial and marine biomes and even small changes in the magnitude and predictability of their environment could markedly influence their future distributions.

## Introduction

To predict how the ranges of species and populations will change through time, we need to understand the mechanisms by which their current distributions are controlled by environmental variability and predictability. In shallow seas, physical variables such as temperature, pH, salinity and wave exposure combine with biological variables such as primary productivity [1] and predation [2] across a variety of temporal and spatial scales to influence organism performance and survival; ultimately dictating species distributions [3]–[5]. Of these factors temperature has a fundamental and pervasive effect on the physiology of ectotherms and is, hence, one of the major physical factors determining species distributions, e.g. [6]. As in general, both the magnitude and variability of habitat temperature vary predictably with latitude and altitude [7], thermal regime is a common correlate of global patterns of biodiversity, e.g. [8], which allows latitudinal comparisons of thermal regime to be used to test for underlying macro-physiological patterns that determine species ranges [8]–[10].

The intertidal zone may be an exception to the latitudinal pattern, as it is an environment where temperature fluctuates over multiple scales. Temperature gradients not only vary with climate and season, but fluctuate regularly during emersion and immersion through each tidal cycle. The strength of these thermal gradients depends on the degree of exposure to solar heating and winter cooling, which varies with latitude, but also with the timing of extreme low waters [3], [11]. Thermal stress in the intertidal zone therefore varies over a variety of spatial and latitudinal scales, which may exceed optimum or even critical temperature limits of animals for short periods of time [12]. The thermal limits of intertidal organisms are therefore likely to be related to temperature at a variety of scales, from rapidly changing microhabitats, to more predictable regional climates. This study aims to compare the thermal tolerance of intertidal limpets alongside measures of their experienced thermal regime to test the scale at which temperature variation can be correlated with temperature limits.

Thermal tolerance can be measured through a wide range of metrics, from biochemical pathways to whole animals, by testing the thermal reaction norm [6], [13]. The range and shape of the resulting thermal dependency varies across latitudes and therefore provides a means for comparing the thermal sensitivity of species and populations across environments, e.g. [14]. The thermal dependency of critical whole animal activities, which rely on the integration of multiple organ systems to maintain tissue energy status and aerobic scope, are likely more sensitive measures with which to assess the physiological tolerance of a species, e.g. [15]–[16]. “Duration tenacity” of limpets has recently been developed as a measure of the thermal dependency of muscular capacity [17]. It measures the time to fatigue of the shell clamping musculature, as it resists a force acting to remove the limpet from the substratum. Duration tenacity was sensitive enough to measure acclimatory shifts in thermal reaction norm after only a 3°C elevation of incubation temperature in the Antarctic *N. Conicnna* [17]. It should therefore be an adequately sensitive measure to detect differences in thermal reaction norm in species across latitude.

This study tests the thermal reaction norms for duration tenacity and survival of four confamilial (Nacellid) limpet species from environments with markedly different temperature means and variation: Antarctic (*Nacella concinna*); temperate New Zealand (*Cellana ornata*); temperate Australia (*C. tramoserica*) and tropical Singapore (*C. radiata*). Duration tenacity was also compared between intertidal and subtidal Antarctic limpets with the subtidal experiment repeated in two summers to assess inter-annual variation. The recorded temperature limits for tenacity were correlated to sea surface temperature data collated from databases and to maximum experienced microhabitat temperature, measured through deployment of temperature loggers (after [18]). The two thermal buffers, warming tolerance and thermal safety margin, were calculated following [19]. These measured the difference in temperature between the upper 50% temperature limit for tenacity (CT_max_; warming tolerance) and the optimum temperature for tenacity (T_opt_; thermal safety margin) and maximum habitat temperature, measured by in situ loggers and from sea surface temperature records. These thermal buffers assess the likely species vulnerability to warming in different aspects of habitat warming [19]) and were used to investigate the scale at which temperature variation was correlated with temperature limits of these four species of Nacellid limpets. Across all measures, tropical *C. radiata,* which experiences the most predictable and least variable environment, was most vulnerable to warming, whilst the two temperate limpets (*C. ornata* and *C. tramoserica*), which live in highly variable and less predictable environments, were the most vulnerable to extreme logger temperatures.

## Materials and Methods

*Nacella concinna* (Strebel 1908) were collected by SCUBA divers from 6 m depth (2007 and 2011) and from low shore bedrock (2011; 0.6–0.8 m above chart datum), at the British Antarctic Survey’s Rothera Research Station (67° 34.25′S, 68° 08.00′W). *Cellana tramoserica* (Holten 1802) were collected from horizontal sandstone exposed in full sun from 0.6–1.0 m above chart datum on 13th beach near Barwon Heads, Melbourne Australia (38° 17.32′S 144° 28.61′E). *Cellana ornata* (Dillwyn 1817) were collected from a boulder strewn shore, 0.8–1.4 m above chart datum, in Dunedin Harbour, New Zealand (45° 49.67′ S, 170° 38.49′ E) outside Portobello Marine Laboratory. *Cellana radiata* (von Born 1778), were collected from man-made breakwaters on St. John’s and Kusu Islands off the South Coast of Singapore from 0.5–2.0 m above chart datum (1° 13.2′N, 103° 51.56′E). All experiments were either conducted in the summer or in typically uniform Singapore tropical weather.

All animals were transported in seawater, in insulated containers to either a recirculating aquarium (Melbourne) or flow through aquaria (elsewhere) and kept at ambient temperature; 0.3±0.1°C at the Rothera Research Station, Antarctica, 13±1.0°C at the Portobello Marine Laboratory, New Zealand, 20.0±0.1°C at the University of Melbourne and 29.0±0.5°C at the Tropical Marine Science Institute, Singapore. Water quality in the Melbourne recirculating aquarium was maintained through both biological and mechanical filtration as well as regular water changes. Animals were held for at least 24 hours before being moved to Perspex jacketed tanks which allowed the seawater temperature to be changed at 0.2±0.1°C per hour, e.g. [20]. Although biomass in each jacketed tank was very low, and there was no visible deterioration in water quality, water was changed regularly with up to 80% of the water being replaced twice daily, with pre-heated/cooled water. No extra food was added to the aquaria but limpets were observed grazing on the tank walls, suggesting that a low level of feeding may have continued throughout the experiments.

Changing temperature at a constant rate, of 0.2±0.1°C per hour, means that temperature limits obtained were the result of both the magnitude and duration of temperature exposure, e.g. [20], which mirrors most natural situations. *N. concinna* were tested between −1.0 and 10.0°C, *C. ornata* between 0.0 and 29.0°C, *C. tramoserica* between −1.5 and 33.6°C and *C. radiata* between 10.6 and 34.6°C (table S1). All tests were conducted with limpets immersed in seawater to avoid any interactive effects of emersion.

Preliminary experiments with each species used different weights, which allowed the identification of a suitable force such that the duration tenacity at ambient temperature ranged mainly between 5 and 30 minutes. The peak force that limpets can resist varies between species [21] and these preliminary trials ensured that the same muscle physiological mechanism, the duration capacity of the muscles responsible for clamping limpets to the substratum, was tested in each species. These weights were 200 g for *N. concinna* and *C. ornata*, 643 g for *C. tramoserica* and 919 g for *C. radiata*. A loop of cotton thread was glued to the shell of Antarctic *N. concinna* and New Zealand *C. ornata* with cyanoacrylate gel before temperatures were changed. The weight required to test the tenacity of temperate *C. tramoserica* and tropical *C. radiata* was heavier than the bonding strength of cyanoacrylate to limpet shells and so a fine soft stainless steel cradle (Leader wire, Bass Pro Shops) was hooked underneath the shell edge, with contact at 4 points around the shell, (modified from [22]), before temperatures were changed. Temperature was then changed and animals were held in seawater at the required experimental temperature for 24 hours before tenacity was measured following Morley et al. [17]. Individuals were tested at one trial temperature only. During all trials any individual that did not have a measurable tenacity was tested by stimulation of the foot with a blunt seeker. Failure of the foot muscle to respond was recorded as functional mortality. This was used to determine upper 50% lethal (UTL) and lower 50% lethal (LTL) limits (following [20]).

To standardise measurements, limpet shells were tapped three times to stimulate a clamping response [22], before the weight was attached. 20 kg mono filament fishing wire was hooked to the stainless steel cradle or cotton loop and passed over two 18KN pulleys (Petzl, France) supported by a retort stand frame. 20 seconds after the limpet shell was tapped, the weight was hooked to the line and gently lowered until the full force was supported directly above the centre of each limpet. The subsequent time taken for the limpet to be pulled from the substratum was recorded.

The three Rothera *N. concinna* trials (subtidal and intertidal in 2 summers) were tested for differences by constructing a gam model [23] and then determining the significance of habitat and year.

The R-package strucchange [24] was used to find the most appropriate piecewise linear breakpoints within the relationship between temperature and duration tenacity. After breakpoint selection, linear models then fitted to the respective sections, incorporating the endpoints in all contiguous sections. Although this created a consistent approach, linear regressions fitted to data below the breakpoint for New Zealand *C. ornata* and Singapore *C. radiata* were re-fitted excluding the breakpoint temperature. New Zealand *C. ornata* also had one extra break point (at 17.5°C) that resulted in a non-significant regression between 14.3 and 17.5°C (Fig. S1) and so this break point was excluded. The break points allowed optimum temperature for duration tenacity (T_opt_) to be identified from which, where possible, the temperature for upper 50% (CT_max_) and lower 50% (CT_min_) tenacity were calculated. Strucchange was also used to calculate the break points for mortality but as mortality typically went from close to 100% survival at one temperature to close to 100% mortality at the next, functional mortality (50% UTL) was therefore quoted as between two temperatures and the average of these two temperatures was used for comparison with habitat temperatures. Data for the upper lethal limit of *N. concinna* was taken from [25].

Micro-habitat temperature was recorded with pre-calibrated temperature loggers, which measure the impact of repeated aerial and aquatic exposure during each tidal cycle. In Melbourne, New Zealand and Singapore two button temperature loggers (SL52T, Signatrol Ltd) were silicone sealed into empty limpet shells and glued to the shore with araldite, where they were deployed for two periods in Melbourne (27/02/08–15/03/08 and 24/03/08–29/03/08), one period in New Zealand (8/12/09–04/01/10) and one period in Singapore (14/04/08–09/06/08). From these temperature records, the maximum temperature during each tidal cycle was determined. Microhabitat temperatures for Rothera were collected by an in-situ temperature logger at 15 m (Rothera Time Series [26]) and button loggers deployed in the intertidal zone [27].

Temperature limits were correlated with experienced microhabitat temperatures recorded by loggers and sea surface temperature records (Table 1). The lowest and highest daily maximum temperatures recorded by loggers were averaged as a measure of the range of maximum microhabitat temperature experienced by limpets through the experimental period. Mean maximum and minimum sea surface temperatures (SST) were collated from database records (Table 1). The upper thermal limits (CT_max_, and UTL) and T_opt_ from the 4 intertidal limpet trials were correlated against these habitat temperatures. To measure the sensitivity of each limpet species to elevated temperature, the buffer of upper 50% (CT_max_) and maximum (T_opt_) temperature for tenacity, above environmental temperature, were calculated as warming tolerance (WT = CT_max_-T_hab_) and thermal safety margin (TSM = T_opt_-T_hab_), where T_hab_ was either mean maximum logger or annual maximum sea surface temperature [19](Table 1).

**Table 1:** Summary of environmental data and thermal sensitivity of limpets from Rothera (*Nacella concinna*), Dunedin New Zealand (*Cellana ornata*), Melbourne Australia (*C. tramoserica*) and Singapore (*C. radiata*).

Annual Sea Surface Temperatures (SST)	Min. SST	Max. SST		WT	TSM	Source
*N. concinna* intertidal	−1.9	1.8		4.1	−0.8	Clarke et al., 2008; RaTS unpub data (1997–2011)
*C. ornata*	7.1	16.1		3.9	−1.8	Shaw et al., 1999 (1953–1997)
*C. tramoserica*	12	21		9.1	−3	http://www.baywx.com/temps.html (2000–2011)
*C. radiata*	27	31		0.2	−3.4	Chou and Lee, 1997
**logger temperatures**	**Lowest summer max.**	**Mean Summer max.**	**Highest summer max.**	**WT**	**TSM**	**Source**
*N. concinna* subtidal	−1.3	−0.1	1.7			RaTS, H. Venables, unpub. Data
*N. concinna* intertidal	−2	0.45	12.3	5.4	0.5	Waller et al., 2006
*C. ornata*	14.1	23.0	37.7	−3	−8.7	
*C. tramoserica*	17.6	27.4	39.3	2.7	−9.4	
*C. radiata*	25.6	32.6	41.2	−1.4	−5	

Warming tolerance, WT = CT_max_-T_hab_, where T_hab_ is the mean maximum daily logger temperature or mean maximum surface seawater temperature. Thermal safety margin, TSM = T_opt_-T_hab,_ where T_hab_ is the same as for WT (after Deutsch et al., 2008). T_opt_, the temperature of maximum tenacity and CT_max_, the upper temperature at which tenacity had dropped to 50%, were taken from Table 2.

† Indicates that the lower limit for functional mortality (LTL) was below the freezing point of seawater.

## Results

### Duration Tenacity

There were differences in length (Table S1) between limpet species (F_5, 417_ = 46.0, p<0.01) but no overall difference in length across temperatures (F_1,417_ = 0.2, p = 0.67) or the interaction between species and temperature (F_5,417_ = 2.2, p = 0.052).

As duration tenacity is a behavioural measure, zero tenacity can either indicate that limpets have reached their physiological limit and can’t resist the force of the weight, or that they choose not to clamp down and carry on moving. Very few zero tenacities were recorded for Rothera *N. concinna*, Melbourne *C. tramoserica* or Singapore *C. radiata* until the limpets reached their thermal limits (Table S1). However, zero tenacities were measured throughout the temperature range of New Zealand *C. ornata*, a limpet which is known to become active when removed from its home scar (M. Barker, pers com), which increased the variability in duration tenacity for *C. ornata*.

**Figure 1:**
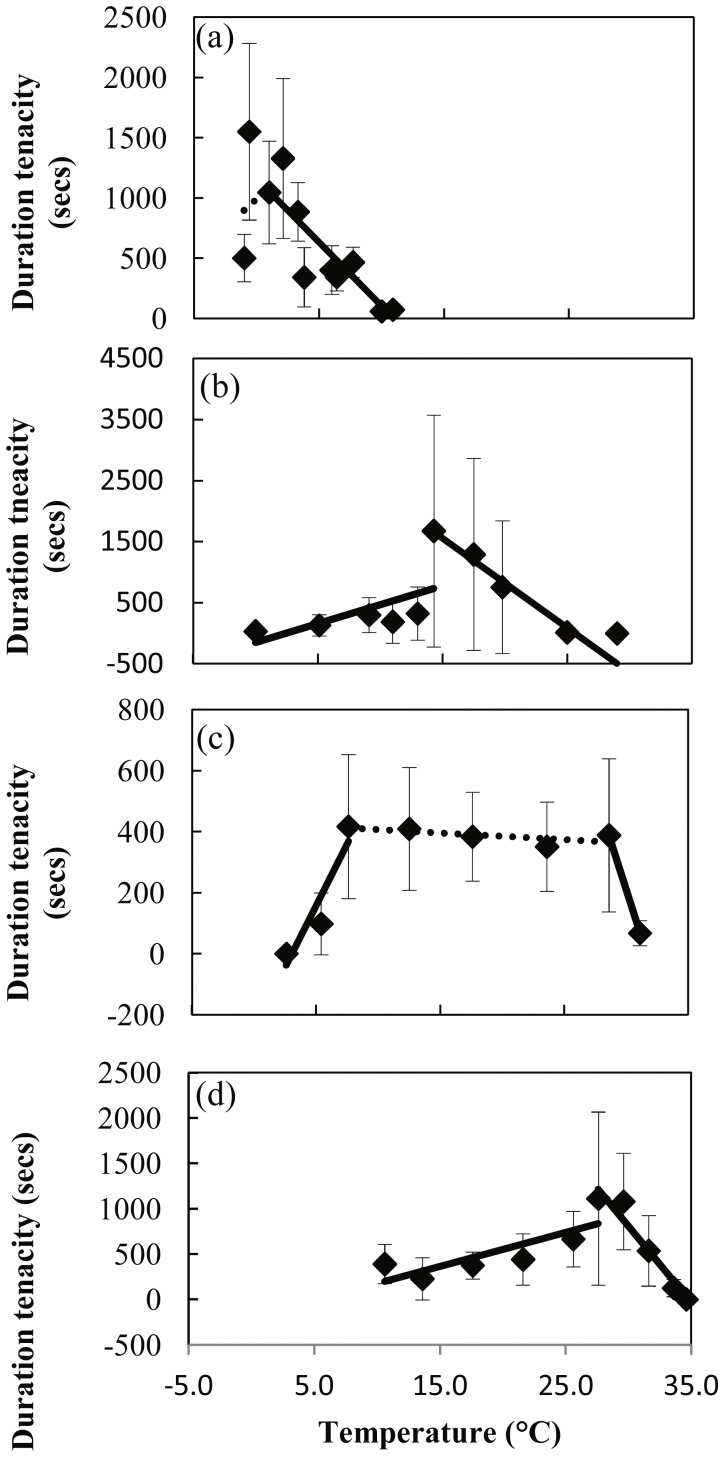
Thermal reaction norm of duration tenacity in Nacellid limpets. Fitted curves are shown. A) Antarctic (*N. concinna*), B) New Zealand (*C. ornata*), C) Melbourne (*C. tramoserica*) and D) Singapore (*C. radiata*). (Mean ±95%CI). Linear regressions are shown with break points calculated using the R-package strucchange (Zeileis et al., 2002).

There was no significant effect of shore height (subtidal versus intertidal zone; t = 0.94, p = 0.35) or year (2007 versus 2011; t = 0.38, p = 0.70), on tenacity of Antarctic *N. concinna*, so these trials were combined (Fig. S2). The thermal reaction norms for duration tenacity shifted to warmer temperatures in warmer environments, from the Antarctic to the tropics (Fig. 1). The pattern of break points was similar for three out of the four species, with *N. concinna* (1.0°C), *C. ornata* (14.3°C) and *C. radiata* (27.6°C) showing a clear T_opt_ for duration tenacity (Fig. 1, Tables 2 and 3). When a linear regression was re-fitted to tenacity values below the breakpoint for *C.* ornata and *C. radiata*, excluding the breakpoint temperature, the relationship with temperature became non-significant. *C. tramoserica* differed with two clear break points, one above and one below a wide temperature range (7.6°C to 28.6°C), over which duration tenacity was relatively constant. T_opt_ for *C. tramoserica* was taken as the average of this range (18.0°C). CT_max_ also increased from the Antarctic to the tropics (Table 2). The sharp change in duration tenacity of *C. ornata* at 14.3°C meant that a CT_min_ could not be calculated for this species. CT_min_ of *N. concinna* could also not be calculated as it is likely to be below the temperature at which seawater freezes.

**Table 2:** Geographic range and thermal limits for limpets from Rothera (*Nacella concinna*), Dunedin New Zealand (*Cellana ornata*), Melbourne Australia (*C. tramoserica*) and Singapore (*C. radiata*).

	Geographic range		Temperature of functional mortality		Temperature of tenacity			
	Southern	Northern	LTL	UTL	CT_min_	T_opt_	CT_max_	Thermal window
*N. concinna*	68°S	54°S	†	12.0	–	1.0	5.9	–
*C. ornata*	41°S	35°S	†	25–29	–	14.3	20.0	–
*C. tramoserica*	43°S	26°S	2.6–5.1	31.1–33.6	5.5	18.0	30.1	24.6
*C. radiata*	35°S	17°N	2.6–4.6	33.6–34.6	21.5	27.6	31.2	9.7

The temperature of 50% upper (CT_max_) and lower (CT_min_) bounds and maximum tenacity (T_opt_) are calculated from the break point and regression fits (Table 2). T_opt_ for *C. tramoserica* was an average value for the temperature range over which tenacity did not change. Geographic ranges taken from http://www.gbif.org/.

**Table 3:** The co-efficients of linear regressions fitted between breakpoints in the relationship between duration tenacity and temperature for limpets from Antarctica (*N. concinna*), Melbourne (*C. tramoserica*), New Zealand (*C. ornata*) and Singapore (*C. radiata*).

	Break point range, °C	Slope ±se	Intercept ±se	F	DF	P
*N. concinna*	−1.0 to 1.0	93.96±161.0	990.67±151.3	0.34	1,108	0.56
	1.0 to 10.9	−105.44±17.0	1159.10±107.2	38.4	1,299	<0.01
*C. ornata*	0.0 to 14.3	61.87±25.4	−152.40±250.1	5.9	1,71	<0.05
	0.0 to 13.0	20.53±13.29	36.7±120.0	2.4	1,62	0.13
	14.3 to 29.0	−146.64±65.5	3759.22±133.1	5.0	1,49	<0.05
*C. tramoserica*	2.6 to 7.6	81.23±20.2	−248.90±113.2	16.1	1,54	<0.01
	7.6 to 28.6	−2.20±5.6	429.31±109.5	0.2	1,93	0.70
	28.6 to 31.1	−128.25±49.9	4056.6±1488.8	6.6	1,35	<0.05
*C. radiata*	10.6 to 27.6	37.46±17.9	−198.87±403.3	4.4	1,76	<0.05
	10.6 to 25.6	22.09±12.63	50.0±266.7	3.0	1,61	0.09
	27.6 to 34.6	−169.24±62.4	5884.64±1909.8	7.4	1,48	<0.01

Breakpoints calculated using the R-package strucchange (Zeileis et al., 2002).

### Functional Mortality

**Figure 2:**
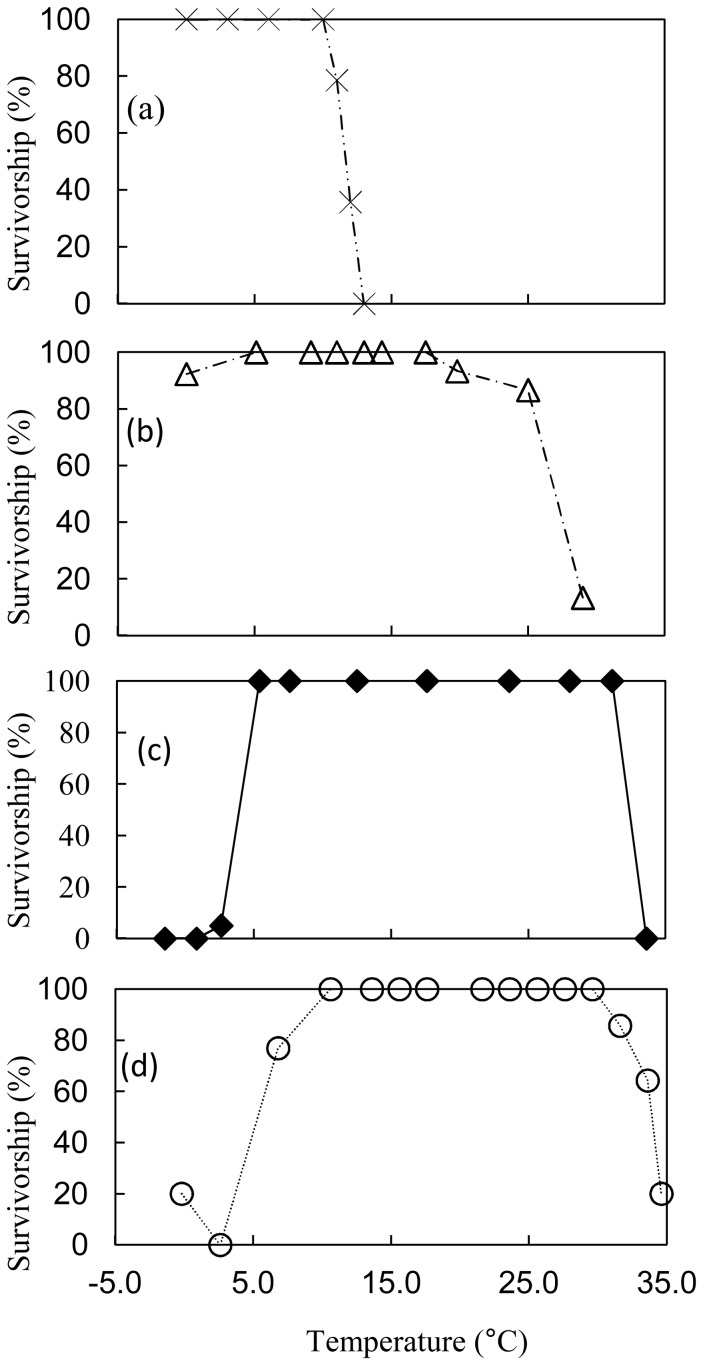
Temperature limits for survival (functional mortality) of A) Antarctic (intertidal *N. concinna*; upper limit from Morley et al, 2009) B) New Zealand (*C. ornata*), C) Melbourne (*C. tramoserica*) and D) Singapore (*C. radiata*) Nacellid limpets (Mean ±95%CI).

The average (50%) UTL increased with environmental temperature from 12.0°C (*N. concinna*) to 25–29°C (27.0°C; *C. ornata*) to 31.1–33.6°C (32.3°C; *C. tramoserica*) and 33.6–34.6°C (34.1°C; *C. radiata*; Fig. 2, Table 2). The difference between CT_max_ and UTL was higher in *N. concinna* (6.1°C) and *C. ornata* (7.0°C), lower in *C. tramoserica* (2.2°C) and *C. radiata* (2.9°C; Table 2). LTL could not be measured in *N. concinna* or *C. ornata* as they survived to below the freezing point of seawater; all but one individual *C. ornata* survived at 0.0°C. The majority of *C. tramoserica* become unresponsive at 2.6°C, 0.8°C and −1.5°C but most limpets recovered when returned to ambient temperature leading to a LTL between 2.6°C and 5.1°C. The LTL of *C. radiata* was between 4.6°C and 2.6°C.

### Microhabitat Temperature

Annual sea surface temperature range was very narrow at both the polar and tropical sites (4°C) but was wider (9°C) at both of the temperate sites (Table 1). Loggers recorded microhabitats with increasing temperatures from the Antarctic to New Zealand to Melbourne to Singapore (Table 1). Logger temperature range was wider in temperate (23.6 and 21.7°C) than in polar (14.3°C) and tropical (15.6°C) latitudes but logger temperature was most unpredictable in the Antarctic and most predictable in the tropics; the co-efficient of variation (CV) of logger temperature was highest in the Antarctic (1.3) than both temperate locations, New Zealand (0.27) and Melbourne (0.19) whilst Singapore had the lowest CV (0.06).

### Comparisons

Even with only 4 species, significant correlations were found between thermal limits and measures of maximum and minimum microhabitat temperature (Table 4). The upper temperature limits T_opt_, CT_max_ and UTL were positively correlated with each other (Table 4). These thermal limits were most consistently correlated with maximum sea surface temperature and the lowest summer maximum logger temperature (Table 4).

**Table 4:** The temperature limits for tenacity and upper lethal limits correlated with environmental temperatures of the four subtidal limpet species.

	Temperature		Maximum tenacity	Upper 50% tenacity	Upper lethal limit
Tenacity	Maximum tenacity		X		
	Upper 50% tenacity	R^2^	**0.95**	X	
		p	**0.05**		
					
Functional mortality	Upper lethal limit	R^2^	**0.95**	**0.99**	X
		p	**<0.05**	**0.01**	
					
Logger temperature	Lowest summer maximum	R^2^	**0.99**	**0.96**	**0.98**
		p	**<0.01**	**<0.05**	**<0.05**
					
	Highest summer Maximum	R^2^	0.91	0.94	**0.98**
		p	0.09	0.06	**<0.05**
					
Sea surface temperature (SST)	min SST	R^2^	**0.97**	0.87	0.86
		p	**<0.05**	0.13	0.14
					
	Max SST	R^2^	**1.0**	**0.95**	**0.96**
		p	**<0.01**	**<0.05**	**<0.05**
					
	Annual SST range	R^2^	0.14	0.36	0.42
		p	0.86	0.64	0.58
					
Latitude	Southern limit	R^2^	0.94	0.91	**0.96**
		p	0.06+	0.09	**<0.05**
					
	Northern limit	R^2^	**0.95**	0.82	0.81
		p	**0.05**	0.18	0.19
					
	Latitudinal range extent	R^2^	0.72	0.55	0.50
		p	0.28	0.45	0.50

Pearson’s correlation co-efficient (R^2^) and significance (p). Significant correlations in bold.

### Upper Temperature Sensitivity

The patterns of warming tolerance (WT), the thermal buffer of CT_max_, and thermal safety margin (TSM), the thermal buffer of T_opt_, were not consistent across species or in relation to different measures of habitat temperature (Table 1). WT above mean maximum sea surface temperature (SST) was highest in *C. tramoserica* (9.1°C) with the lowest WT (0.2°C) in *C. radiata*. TSM above SST declined with decreasing latitude from the Antarctic to the equator (−0.8°C for *N. concinna* to −3.4°C for *C. radiata*; Table 1). Mean summer maximum logger temperatures gave different patterns for WT and TSM. *N. concinna* had the highest WT (5.4°C), with a negative WT for both *C. ornata* and *C. radiata* (−3.0 and −1.4°C respectively). Both temperate limpets, *C.ornata* and *C. tramoserica* had the lowest TSM (−8.7 and −9.4°C respectively). *N. concinna* also had the highest TSM.

## Discussion

There were clear temperature effects on the duration tenacity (CT_min_, T_opt_ and CT_max_) and upper lethal limits (UTL) that differed between the four species of limpets. Furthermore these could be correlated with elements of their experienced thermal environments.

### Duration Tenacity and Temperature

Duration tenacity measures the time of constant (tetanic), fixed muscle length (isometric), contraction to the point of muscle fatigue when the limpet can no longer hold on. Fatigue during isometric tetanic contraction has largely been investigated in skeletal muscle and, whilst a number of biochemical changes have been associated with muscle fatigue, key mechanisms include the dephosphorylation of ATP and creatine phosphate (CrP) and the resultant build up of metabolic end-products, particularly inorganic phosphate [28], [29]. Most invertebrate groups, including molluscs, use phospho-L-arginine (PLA) instead of CrP [30] and both PLA and ATP concentrations have been shown to reduce as limpets approach their upper temperature limits [31]. It is probable that a portion of the energetic support for tenacity comes from anaerobic metabolism, as is found in other bivalve adductor muscles, e.g. [32]. However, even if anaerobic metabolism is important, adductor muscle activity utilises ATP, and so initial muscle tissue energy status and metabolic capacities will still be linked to muscle fibre aerobic capacities, which are expected to decline at extremes of temperature [32], [33].

The gradual reduction in tenacity at temperatures above an optima fits with the principle that routine costs are increasing as limpets warm up, reducing muscle tissue energy status. However, only the thermal dependency of tenacity in Melbourne *C. tramoserica* was close to a typical rate temperature shaped curve [34], with an optimal range and clearly defined upper and lower temperatures, beyond which, duration tenacity reduced. The wide temperature range over which the duration tenacity of *C. tramoserica* was constant (7.6 to 28.6°C) matches with the temperature response of species that experience variable thermal environments and therefore have wide thermal optima [9]. The temperature dependence of force exerted during isometric contraction largely depends on the combination of tetanic and twitch tension, which are relatively independent of temperature over an organism’s optimum temperature range [35]. The sharp decline in duration tenacity above and below this range is likely caused by a drop in phosphorylation of ATP and PLA at extreme temperatures.

There was a different relationship between duration tenacity and temperature for *C. ornata* and *C. radiata*, which were both best described by separate relationships either side of a single optimum. For these two species there was also a distinct drop in duration tenacity below the optimum and a much shallower linear decrease in tenacity, or even temperature independence, below this drop. At low temperatures the ability to maintain foot shape creates suction, and mucus provides some adhesion, even at temperatures where muscular capacity was negligible [22]. This residual tenacity is not expected to be greatly affected by temperature.

However, this does not explain why the breakpoint for duration tenacity in *C. ornata* and *C. radiata* occurs at temperatures within their normally experienced range (14.2 and 27.6°C respectively). Factors other than aerobic scope are therefore likely to be affecting the temperature response of duration tenacity.

### Environmental Temperature Correlations

One of the predicted outcomes of a general warming of the earth’s surface is an increase in extreme events and anomalies [36]. Depending on the duration of exposure, extreme stress events may be more important in defining thermal limits than mean environmental temperatures [37], [38]. For example, marine ectotherms that live in regions affected by irregular events, such as El Niño, do not often have the physiological plasticity to cope with these episodic extreme temperatures [39]. It is, therefore, important to understand the scale of variation in the physical environment that an organism responds to, particularly in the intertidal zone where species are exposed to both aerial and aquatic environments [40].

The thermal reaction norms for duration tenacity and upper lethal limits among all four limpet species were shifted towards higher temperatures in warmer environments, and, although the correlations of only 4 species must be interpreted with caution, were most consistently correlated with aspects of both maximum sea surface temperature (SST) and microhabitat (biomimetic logger) temperature. The pattern of thermal sensitivity (WT and TSM) between species also differed between comparisons with SST and those with loggers. Loggers record the effect of both water and air temperature on limpet body temperature and therefore capture more of the environmental variability experienced by the limpets. Despite this, there was a latitudinal trend of reducing TSM for more northerly limpet species, indicating that all limpet species are subject to periods when seawater temperatures are above their optimal temperature for tenacity.

Tropical limpets had the lowest WT (relative to SST) agreeing with previous studies that suggest the thermal limits of tropical ectotherms are already close to their mean maximum environmental temperatures and therefore have limited capacity to cope with further warming [18], [41]–[43]. *C. radiata* also had negative TSMs (relative to logger temperature), indicating that they are also sensitive to the combined effect of water and air temperature. The intertidal zone has some of the highest spatial and temporal heterogeneity of temperature [40] and daily variation between microhabitat temperature on the shore can be higher than the variation in mean climate values over many degrees of latitude [44]. If this temperature variation is unpredictable, then thermal acclimatisation may not provide an energetic advantage disrupting seasonal thermal correlations with climate [45], [46]. Australian *C. tramoserica* and New Zealand *C. ornata* live in highly variable environments where summer temperatures change rapidly and unpredictably. Summer air temperature in Melbourne can vary from 4.5 to 46.4°C (http://www.weatherzone.com.au/climate/station.jsp?lt=site&lc=86071 (1908–2008)) and in Dunedin New Zealand from 4.3 to 29.9°C (http://cliflo.niwa.co.nz) which can result in a much larger daily than seasonal change in temperature, e.g. [46](Sinclair et al. 2006). Such variation may explain the limited physiological capacity of *C. ornata* and *C. tramoserica* to respond to extreme temperatures which was reflected in their negative TSMs. This lack of physiological capacity to respond to unpredictable temperatures may also apply to adaptive capacities, which are surprisingly rapid in some species [47], [48].

Whilst Antarctic summer intertidal logger temperatures had the highest co-efficient of variation (1.3) there are predictable differences between summer and winter temperature. Monthly air temperature is much less variable in summer, ranging between 7 and −5°C, whilst winter air temperatures are predictably colder and more variable, ranging between 3 and −23°C (BAS meteorological records). The seasonal variation in their environment is sufficiently predictable for intertidal *N. concinna* to exhibit seasonal physiological acclimation [49]. Living in a variable but predictable environment may explain the high WT and TSM of intertidal *N. concinna* in relation to mean summer maximum logger temperature.

The duration of exposure to elevated temperatures during low water will, however, be less than the 24-hour treatment used in the current study, and temperature limits have been found to be higher in experiments using cyclic, rather than constant temperatures [50]. Many animals, including some intertidal molluscs, utilise metabolic depression as a strategy to survive short periods of extreme heat, e.g. [51], which would extend their survival time at high temperatures.

Despite this, geographic comparisons of duration tenacity and lethal limits of 4 Nacellid limpets have shown that a combination of both sea and air temperature variation may affect the distribution of intertidal species. If extreme events do become more common in mid-latitudes, then temperate species may become more vulnerable to the effects of climate change than those from more stable low latitude environments. The thermal reaction norm of duration tenacity does not follow the curve expected for an aerobic physiological response in all species and the mechanisms underlying muscle thermal physiology may therefore be another important factor that might determine limpet response to climate change.

## Supporting Information

Figure S1 **Thermal reaction norm of duration tenacity of New Zealand** ***C. ornata*** **with two break points.** 0 to 14.3°C, F_1,71_ = 5.9, p<0.05; 14.3 to17.5°C, F_1,20_ = 0.12, p = 0.73; F_1,40_ = 3.3, p = 0.08. Mean ±95% CI.
(DOC)

Figure S2 **Thermal reaction norms for duration tenacity of the 3** ***N. concinna*** **trials.** Mean ±95% CI
(DOC)

Table S1 **The number and lengths of Nacellid limpets tested at each temperature.**
(DOC)
