# Supplementary figures and images for: Thermal Reaction Norms and the Scale of Temperature Variation: Latitudinal Vulnerability of Intertidal Nacellid Limpets to Climate Change
Source: PLoS One. 2012 Dec 21;7(12):e52818. doi: 10.1371/journal.pone.0052818 (PMC3528710; doi:10.1371/journal.pone.0052818)

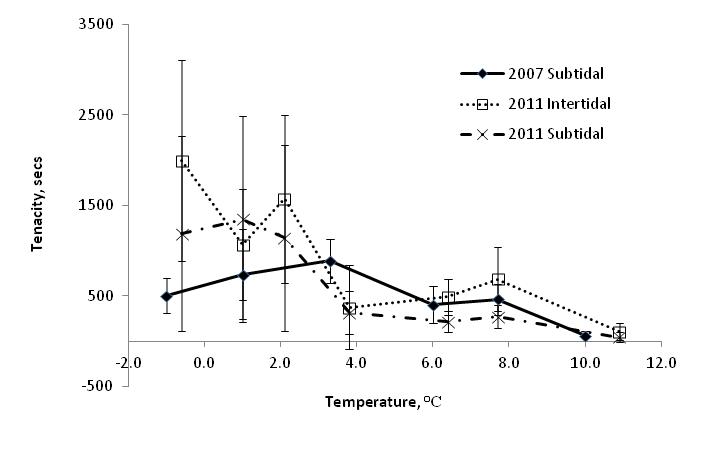

Supplement: Figure S2 — Thermal reaction norms for duration tenacity of the 3 N. concinna trials. Mean ±95% CI (DOC) [file pone.0052818.s002.doc]
