# Supplementary material for: Thermal Reaction Norms and the Scale of Temperature Variation: Latitudinal Vulnerability of Intertidal Nacellid Limpets to Climate Change
Source: PLoS One. 2012 Dec 21;7(12):e52818. doi: 10.1371/journal.pone.0052818 (PMC3528710; doi:10.1371/journal.pone.0052818)
Supplement: Table S1 — The number and lengths of Nacellid limpets tested at each temperature. (DOC) [file pone.0052818.s003.doc]

| Location | trial | Species | Temperature  /°C | Length  mean±SE | n | zero tenacity/n |
| --- | --- | --- | --- | --- | --- | --- |
| Rothera | 2007 subtidal | *N. concinna* | -1.0 | 28.0±1.0 | 25 | 0 |
|  |  |  | 1.0 | 30.9±1.9 | 21 | 0 |
|  |  |  | 3.3 | 30.1±1.5 | 26 | 0 |
|  |  |  | 6.0 | 30.8±1.5 | 17 | 0 |
|  |  |  | 7.7 | 31.5±1.4 | 26 | 0 |
|  |  |  | 10.0 | 32.1±1.6 | 27 | 10 |
| Rothera | 2011 subtidal | *N. concinna* | -0.6 | 27.2±1.5 | 10 | 1 |
|  |  |  | 1 | 24.3±0.92 | 18 | 1 |
|  |  |  | 2.1 | 23.8±1.0 | 13 | 0 |
|  |  |  | 3.8 | 23.7±0.85 | 11 | 1 |
|  |  |  | 6.4 | 21.1±0.93 | 11 | 1 |
|  |  |  | 7.7 | 25.7±0.95 | 15 | 0 |
|  |  |  | 10.9 | 24.6±1.4 | 14 | 1 |
| Rothera | 2011 intertidal | *N. concinna* | -0.6 | 26.8±1.6 | 9 | 0 |
|  |  |  | 1 | 24.5±0.84 | 24 | 0 |
|  |  |  | 2.1 | 23.8±1.2 | 11 | 0 |
|  |  |  | 3.8 | 22.0±1.1 | 14 | 0 |
|  |  |  | 6.4 | 21.4±0.99 | 13 | 0 |
|  |  |  | 7.7 | 23.0±0.95 | 14 | 0 |
|  |  |  | 10.9 | 21.3±2.1 | 9 | 4 |
| New Zealand | 2010 intertidal | *C. ornata* | 0 | 25.7±1.3 | 13 | 5 |
|  |  |  | 5.1 | 23.5±1.4 | 13 | 6 |
|  |  |  | 9.1 | 24.9±1.0 | 13 | 1 |
|  |  |  | 11 | 24.0±1.5 | 14 | 9 |
|  |  |  | 13 | 23.8±1.9 | 13 | 6 |
|  |  |  | 14.3 | 22.8±2.3 | 12 | 3 |
|  |  |  | 17.5 | 23.5±1.7 | 13 | 7 |
|  |  |  | 19.8 | 22.9±1.7 | 15 | 5 |
|  |  |  | 25 | 23.0±1.7 | 17 | 11 |
|  |  |  | 29 | 21.8±1.1 | 15 | 2 |
| Australia | 2008 intertidal | *C. tramoserica* | -1.5 | 25.7±1.1 | 21 |  |
|  |  |  | 0.8 | - | 20 |  |
|  |  |  | 2.6 | 29.0±1.1 | 20 | 18 |
|  |  |  | 5.4 | 29.0±1.4 | 18 | 10 |
|  |  |  | 7.6 | 30.0±1.4 | 19 | 0 |
|  |  |  | 12.5 | 27.8±1.2 | 18 | 2 |
|  |  |  | 17.6 | 28.5±1.2 | 20 | 0 |
|  |  |  | 23.6 | 27.3±1.5 | 19 | 1 |
|  |  |  | 28.6 | 26.7±1.3 | 19 | 0 |
|  |  |  | 31.1 | 27.5±1.3 | 19 | 5 |
|  |  |  | 33.6 | 28.1±1.2 | 19 |  |
| Singapore | 2008 intertidal | *C. radiata* | -0.2 | - | 9 |  |
|  |  |  | 6.8 | - | 13 |  |
|  |  |  | 10.6 | 37.2±0.62 | 10 | 0 |
|  |  |  | 13.6 | 37.2±1.5 | 5 | 0 |
|  |  |  | 17.6 | 34.3±1.1 | 7 | 0 |
|  |  |  | 21.6 | 31.6±0.81 | 16 | 2 |
|  |  |  | 25.6 | 33.4±0.90 | 25 | 2 |
|  |  |  | 27.6 | 31.4±0.86 | 18 | 2 |
|  |  |  | 29.6 | 36.4±0.93 | 11 | 0 |
|  |  |  | 31.6 | 35.1±1.2 | 16 | 0 |
|  |  |  | 33.6 | 34.2±1.4 | 13 | 1 |
|  |  |  | 34.6 | 31.9±1.9 | 19 | 3 |
