# Supplementary material for: Thermal Reaction Norms and the Scale of Temperature Variation: Latitudinal Vulnerability of Intertidal Nacellid Limpets to Climate Change
Source: PLoS One. 2012 Dec 21;7(12):e52818. doi: 10.1371/journal.pone.0052818 (PMC3528710; doi:10.1371/journal.pone.0052818)
Supplement: Supplementary file 3 [file pone.0052818.s001.doc]

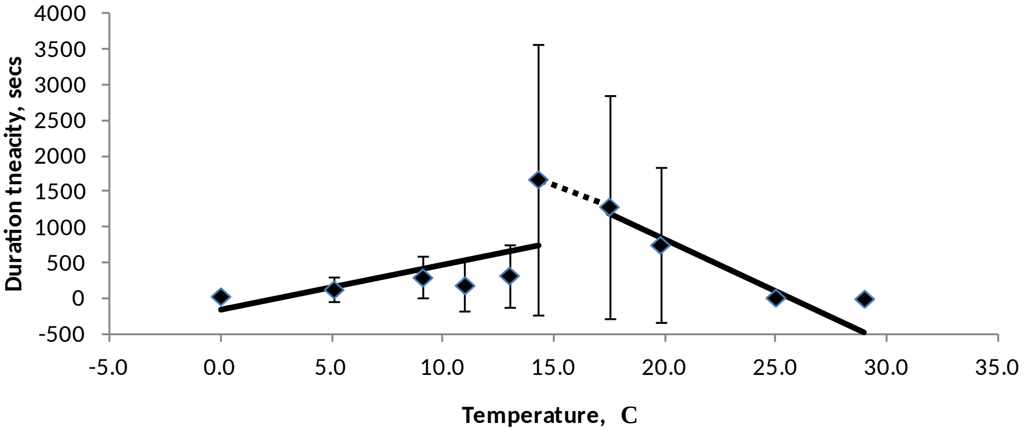
Duration tneacity, secs
4000
3500
3000
2500
2000
1500
1000
500
0
-500
-5.0
0.0
5.0
10.0
15.0
20.0
25.0
30.0
35.0
Temperature, C
